# Effects of Polysaccharides Extracted from Stem Barks on the Spontaneous Contractile Activity of the Ileal Smooth Muscle

**DOI:** 10.3390/molecules30153156

**Published:** 2025-07-28

**Authors:** Ericka Lorleil Mayindza Ekaghba, Olivier Perruchon, Patrice Lerouge, Line Edwige Mengome

**Affiliations:** 1 Institut de Pharmacopée et Médecines Traditionnelles (IPHAMETRA), Centre National de la Recherche Scientifique et Technique (CENAREST), Libreville BP 12 141, Gabon; ericka.mayindza@iphametra.org; 2 Université de Rouen Normandie (UNIROUEN), Normandie University, GlycoMEV UR 4358, SFR Normandie Végétal FED 4277, Innovation Chimie Carnot, IRIB, GDR CNRS Chemobiologie, RMT Bestim, F-76000 Rouen, France; olivier.perruchon1@univ-rouen.fr

**Keywords:** pectin, hemicellulose, glucuronoarabinoxylan, spasmolytic activity, spasmogenic activity

## Abstract

Decoctions of stem barks from *Aucoumea klaineana*, *Canarium schweinfurthii*, *Pentadesma butyracea* and *Scorodophloeus zenkeri* are used against affections of irritable bowel syndrome in Gabonese traditional medicine. In the present study, we aim to determine whether the bark polysaccharides may contribute to the activity of these plants against the symptoms of gastrointestinal disorders. To this end, we investigated the structure and the pharmacological activity of polysaccharides extracted from their stem barks. The pectic and hemicellulose polysaccharides were isolated, and their sugar compositions were determined by gas chromatography. In addition, analysis by MALDI-TOF mass spectrometry of oligosaccharides released after digestion with an endo-xylanase indicated that glucuronoarabinoxylans are the main hemicellulose of stem barks. We then evaluated the influence of the polysaccharide fractions on the spontaneous contractile activity of rat ileal smooth muscle and the cholinergic system. Spasmolytic activity of pectic fractions from all stem barks, as well as lemon polygalacturonic acid, were observed, indicating that these extracts exhibit a myorelaxant activity. In contrast, the bark hemicellulose fractions, as well as commercially available beechwood glucuronoxylan and wheat arabinoxylan, were demonstrated to be able to increase the basal contractile activity of smooth muscle. These data show that, beyond physicochemical effects affecting the bowel water content, plant polysaccharides have also an impact on the spontaneous smooth muscle contractility, the main mechanism involved in the pathophysiology of gastrointestinal disorders.

## 1. Introduction

In recent years, polysaccharides isolated from various natural sources have attracted increasing attention due to their wide variety of pharmacological properties, such as antitumor, immunomodulatory, antioxidant and anti-inflammatory activities. For instance, arabinogalactan and galactomannan from plants, β-glucans from fungi and sulfated polysaccharides from algae were shown to possess antioxidant and immunomodulatory activities [1]. Pectic polysaccharides also exhibit pharmacological properties, such as immunoregulatory, anti-inflammatory, hypoglycemic, antibacterial, antioxidant and antitumor activities [2].

The extracts prepared from the stem barks of trees are widely used in traditional medicine by the African population to answer their health care needs. For instance, stem bark decoction or maceration from *Aucoumea klaineana*, *Canarium schweinfurthii*, *Pentadesma butyracea* and *Scorodophloeus zenkeri* are used in Gabon against the symptoms of irritable bowel syndrome [3]. In a previous study, we demonstrated that bioflavonoids from the decoction of *P. butyracea* stem barks exhibited an antidiarrheal activity [3]. The barks, part of the woody biomass, contain many water-extractible metabolites that are responsible for most pharmaceutical activities used in traditional medicine. In addition, barks also contain lignin, starch, cellulose, hemicelluloses and pectins. Regarding the two latest polysaccharide families, only a few studies have described the structure of these cell wall polymers [4,5,6,7,8]. The main hemicelluloses isolated from barks were reported to be xylans, and their substituted forms, arabinoxylans and glucuronoarabinoxylans, which share a common poly β(1-4)-linked xylose (Xyl) backbone. Pectins from barks are acidic polymers containing galacturonic acid (GalA) units that mainly gather homogalacturonan and rhamnogalacturonan I (RG-I). Homogalacturonan is a linear polymer consisting of α(1-4)-linked GalA residues that can be partially *O*-methylesterified and *O*-acetylated. RG-I is composed of a backbone of [→4-D-GalAα→2-L-Rhaα1→] repeating motifs with rhamnose (Rha) residues substituted at *O*-4 with a wide range of arabinan, galactan and arabinogalactan side-chains. The aim of the present study is to characterize the polysaccharides of stem barks of *A. klaineana*, *C. schweinfurthii*, *P. butyracea* and *S. zenkeri* and to investigate their pharmacological activity against the affections of irritable bowel syndrome. To this end, the structure of polysaccharides extracted from stem barks was determined and their spasmolytic or spasmogenic activities on the ileal smooth muscle were evaluated: two mechanisms involved in the pathophysiology of gastrointestinal disorders.

## 2. Results

### 2.1. Structural Identification of Polysaccharides from Barks

The polysaccharides from stem barks of *A. klaineana*, *C. schweinfurthii*, *P. butyracea* and *S. zenkeri* were extracted by successive treatments with ammonium oxalate and 1 M and 4 M potassium hydroxide (KOH). Ammonium oxalate is widely used for the extraction of pectins, whereas KOH enables the solubilization of hemicelluloses from the remaining cell wall residues. As reported in Table 1, the pectic fractions, named oxa, only accounted for 1–5% of polysaccharides extracted from barks. In contrast, hemicellulose fractions, named, respectively, K1 and K4 for samples extracted with 1 M and 4 M KOH, represented about 95–98% of the solubilized polysaccharide fractions. This large predominance of hemicelluloses in barks was previously reported [6,8].

The sugar composition of pectic extracts were determined by analysis of trimethylsilyl derivatives of their constitutive monosaccharides using gas chromatography with a flame ionization detector (GC-FID) (Table 2). As expected for the pectic extracts, arabinose (Ara), Rha, galactose (Gal) and GalA were the main monosaccharides identified in these fractions. Because homogalacturonan and RG-I backbones are composed of, respectively, repeating units of [→4-D-GalAα1→] and [→4-D-GalAα→2-L-Rhaα1→], the [GalA/Rha] ratio indicates the relative percentage of these two domains in pectin extracts. Table 2 shows that homogalacturonan mainly dominates in pectins extracted from *S. zenkeri* ([GalA/Rha] = 4.23) and from *P*. *butyracea* ([GalA/Rha] = 2.25). In contrast, in *A. klaineana* and *C. schweinfurthii* oxalate extracts, the [GalA/Rha] ratio is about one, which demonstrates that RG-I is the main pectic polymer in stem barks of these two plants. In addition, the [GalA/Rha] ratio ranging from 0.75 to 2.12 in oxalate extracts indicates the relative ratio of galactan and arabinan side-chains in RG-I, and the [(Gal+Ara)/Rha] ratio shows the degree of substitution of the RG-I backbone. It is noteworthy that the detection by GC-FID analyses of Xyl and mannose (Man) in the pectic fractions indicates that low amounts of mannans and xylans were co-extracted with pectins.

The sugar composition of KOH fractions shows that hemicellulose extracts are enriched in Xyl, with Ara and glucose (Glc) present to a lesser extent (Table 3). These data suggest that arabinoxylans are the main hemicelluloses present in stem barks of the four plants. Moreover, the Xyl/Ara ratio shows that the relative abundance of Ara substitution on the xylan backbone in K1 and K4 extracts ranges from 2 (highly branched xylans) to 10 (weakly branched xylans).

**Figure 1:**
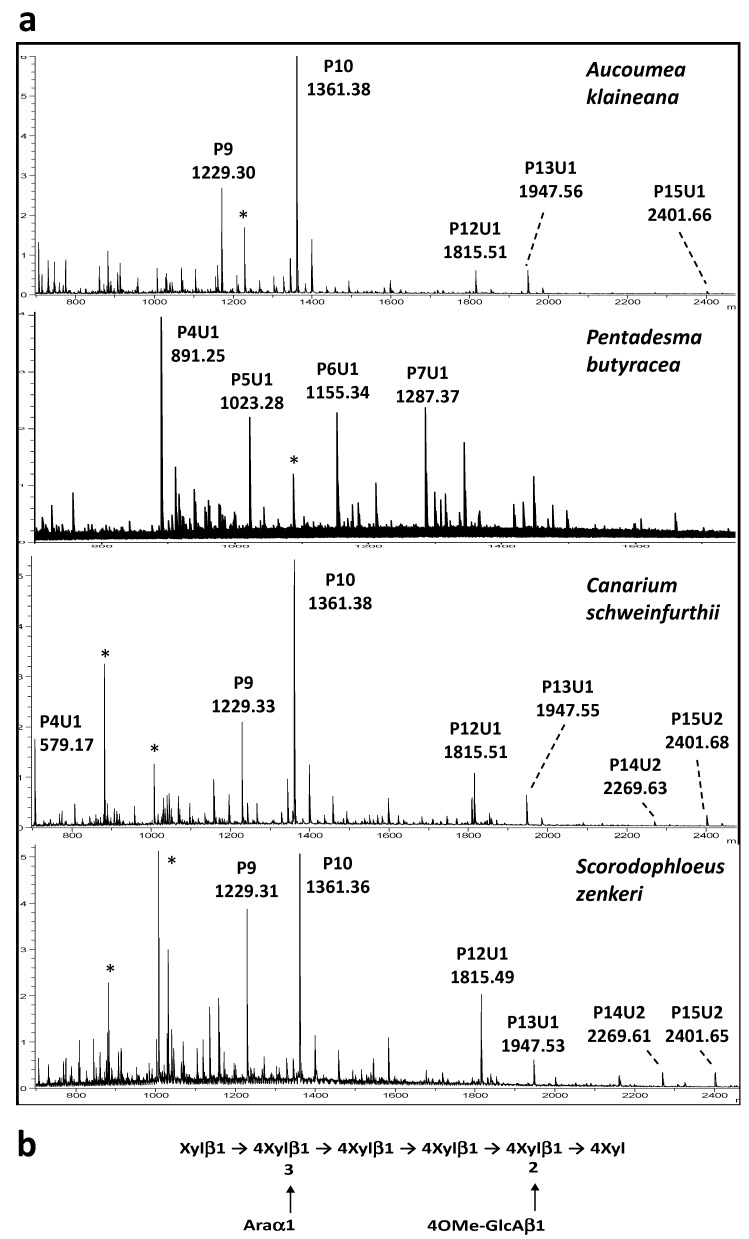
(**a**) MALDI-TOF mass spectra of the endo-xylanase-digested K4 hemicellulose fractions isolated from stem barks of *A. klaineana*, *C. schweinfurthii*, *P. butyracea* and *S. zenkeri*. PnUm: glucuronoarabinoxylan fragments containing *n* pentoses (P) and m 4-*O*-methyl glucuronic acid residues (4OMe-GlcA, U for uronic acid). *: unidentified ions. (**b**) the proposed structure of the repeating oligosaccharide unit of glucuronoarabinoxylans of stem barks.

It should be noted that the detection of Glc residues in the hemicellulose fractions suggests that xyloglucans may also contribute to the hemicellulose content. As a consequence, to get complementary information on the structure of the K1 and K4 hemicellulose fractions, these extracts were submitted to digestion with endo-glycosidases that are able to cleave either the xylan backbone of arabinoxylans and glucuronoarabinoxylans or the glucan backbone of xyloglucans. The resulting generated oligosaccharides were then analyzed by MALDI-TOF mass spectrometry (MALDI-TOF MS). To investigate the putative presence of xyloglucan in K1 and K4 extracts, hemicellulose fractions were first digested with an endo-glucanase that is able to cleave the xyloglucan backbone after unsubstituted β(1-4)-linked Glc units, releasing short oligosaccharides. No oligosaccharide was detected by MALDI-TOF MS in the endo-glucanase digests, suggesting that the hemicellulose fractions did not contain detectable amounts of xyloglucans. Thus, we concluded that the Glc content detected in the hemicellulose fractions may originate from contaminating starch or other glucans. In contrast to the endo-glucanase treatment, oligosaccharides were identified by MALDI-TOF MS analysis of the endo-xylanase-digested K1 and K4 fractions. This enzyme is able to cleave after unsubstituted β(1-4)-linked Xyl residues of the xylan backbone, generating oligosaccharides of various sizes depending on the backbone substitution with arabinosyl residues. It is important to point out that plant xylans may also be substituted by 4-*O*-methyl glucuronic acid (4OMe-GlcA) to yield glucuronoarabinoxylan. MALDI-TOF MS profiles of endo-xylanase-digested hemicellulose fractions show a mixture of [M+Na]^+^ ions assigned to pentose oligomers, noted Pn, with *n* being the number of Ara or Xyl residues, which share the same molecular mass (Figure 1a). Xylan fragments containing 4OMe-GlcA units (noted U for uronic acid) were also detected in the MS spectra. These oligosaccharide fragments differ by 190 mass units from pentose oligomers, as illustrated for the spectra of K4 fractions, represented in Figure 1a. MALDI-TOF MS profiles of K1 and K4 fractions mainly differ by the relative intensity of oligosaccharide fragments, which confirms that stem barks contain glucuronoarabinoxylan populations differing in the level of Ara substitution.

On the basis of the sugar composition, MALDI-TOF MS profiles of endo-xylanase-digested polymers and the data from the literature on plant hemicelluloses, we conclude that the polysaccharide fractions extracted from barks with potassium hydroxide are mainly glucuronoarabinoxylan composed of the repeating oligosaccharide units, represented in Figure 1b. The level of Ara substitution is the main variable observed between bark samples and between 1 M and 4 M KOH fractions.

### 2.2. Activity of Stem Bark Polysaccharides on the Contractile Activity of Smooth Muscle

Gastrointestinal disorders are related to intestinal motility disturbances. Treatment of such disorders requires the prescription of drugs exhibiting either spasmolytic or spasmogenic activities on the intestinal contractile activity. As a consequence, the impact of pectic and hemicellulose fractions on the spontaneous contractile activity of rat ileal smooth muscle was assessed in vitro, as previously reported for plant metabolites [3].

#### 2.2.1. Spasmolytic Activity of Pectin Fractions

The activity of pectic fractions on the contractile activity were tested on fragments of iliac smooth muscle isolated from Wistar rats. Table 4 and Figure S1 show that these fractions induced an inhibition of their spontaneous contractile activity. For comparison, we also tested loperamide, an antidiarrheic molecule, and lemon polygalacturonic acid, a de-esterified commercially available homogalacturonan containing about 30% of RG-I. The inhibition of the spontaneous contractile activity of excised ileum fragments with PBoxa and SZoxa (59% and 52.5%) was comparable to loperamide (55%). In contrast, this smooth muscle relaxation reaches 100% after addition of AKoxa and CSoxa fractions, as well as with lemon polygalacturonic acid (Table 4 and Figure S1). It therefore appears that the pectic fractions exhibit spasmolytic activity.

#### 2.2.2. Effects of Pectin Fractions on the Cholinergic System

To investigate the effect of pectic fractions on the cholinergic system, PBoxa and SZoxa, two pectic extracts that were obtained in sufficient amounts to perform these bioassays, were applied to ileum fragments after pre-contraction induced by acetylcholine, a chemical neurotransmitter. The percentage of relaxation induced by these two fractions on the pre-contracted smooth muscle were 41.9 ± 4% and 59.1 ± 9%, respectively (Table 4). This indicates that these pectic fractions are, therefore, able to antagonize the stimulating action of acetylcholine, thus suggesting that these two extracts are antispasmodic. However, these assays were performed on only two pectic fractions, and as a consequence, more experiments have to be carried out on other pectin samples to draw definitive conclusions.

#### 2.2.3. Spasmogenic Activity of Hemicellulose Fractions

The impacts of hemicellulose fractions on the spontaneous contractile activity of smooth muscle fragments were also investigated. Other commercially available polysaccharides, such as tamarind xyloglucan, beechwood glucuronoxylan and wheat arabinoxylan, were also tested (Table 5). These polymers are representative of the main classes of plant hemicelluloses. Xyloglucan from tamarind seeds is composed of a β(1-4)-linked glucan backbone that is substituted by α(1-6)-linked Xyl on three consecutive Glc units, the fourth residue being unsubstituted. Additional substitutions of Xyl with β(1-2)-linked Gal also occur. Wheat arabinoxylan is composed of a β(1-4)-linked xylan backbone substituted by α(1-3)-linked and α(1-2)-Ara residues. Finally, the xylan backbone of beechwood glucuronoxylan is substituted with 4OMe-GlcA. Picosulfate, a laxative molecule used for the treatment of constipation, was tested as a positive control. The results showed that the hemicellulose K1 and K4 fractions isolated from the four plants induce a very large increase in basal contractile activity, with the highest stimulation observed with the K4 fractions (Table 5 and Figure S1). Commercially available hemicelluloses also induced the stimulation of spontaneous contractile activity of smooth muscle fragments (Table 5). In contrast, tamarind xyloglucan did not induce any stimulation suggesting that the spasmogenic activity depend on the hemicellulose structure.

## 3. Discussion

Dietary polysaccharides are important sources of energy and prebiotic oligosaccharides [9]. In addition, they exert major impacts on symptoms of the irritable bowel syndrome [10]. For instance, pectin is usually considered as an antidiarrheic polymer because it is a water-*soluble* dietary *fiber* that can absorb water and fat, thus leading to the increase of the stool consistency in the intestinal tract and the reduction of transit time [11]. In contrast, hemicelluloses belong to insoluble fibers that have a mechanically irritating effect on the bowel mucosa that induces the secretion of water and, as a consequence, hemicelluloses exhibit laxative activities [12]. It should be noted that, beyond these physicochemical effects affecting the bowel water content, the activity of polysaccharide on smooth muscle contractibility has received very little attention to date [13,14].

The stem barks investigated in the present study are commonly used in Gabonese traditional medicine to alleviate the symptoms of irritable bowel syndrome. Indeed, decoctions of stem barks from *A. klaineana* and *P. butyracea* have antidiarrheal activities, whereas the one from *C. schweinfurthii* is used in traditional medicine against intestinal pain. Moreover, the decoction of stem barks of *S. zenkeri* is used as a laxative [3]. In the present study, we have investigated whether the polysaccharides isolated from these barks may contribute to the beneficial activity of these plants on diarrhea or constipation by acting on the smooth muscle’s contractility, a main mechanism involved in the pathophysiology of gastrointestinal disorders [15].

To get information on their polysaccharide content, stem barks were first submitted to treatments with hot water, methanol and ethanol to remove weakly associated polymers or bioactive metabolites, such as phenolic compounds, of which some were shown to be responsible for the biological activity of stem bark decoctions on the symptoms of irritable bowel syndrome [3]. Polysaccharides were then isolated from the insoluble residues of the four plants by sequential extractions with ammonium oxalate and KOH. This first enabled the isolation with oxalate of low amounts of pectin, not exceeding 5% of the total polymers. Then, treatments with 1 M and 4 M KOH allowed the solubilization of hemicelluloses (>95%). Pectic and hemicellulose sugar compositions of the different stem barks were similar. Sugar compositions of the oxalate fractions indicated that these extracts are mainly composed of homogalacturonan and RG-I in various proportions, as indicated by their [GalA/Rha] ratio. Hemicellulose fractions are enriched in Xyl residues, and the analysis by MALDI-TOF mass spectrometry of oligosaccharides released after digestion with an endo-xylanase shows that they are mainly glucuronoarabinoxylans with various levels of Ara and 4OMe-GlcA substitutions. Acetylated glucuronoxylans have previously been reported in *A. klaineana* wood [16]. Moreover, glucuronoarabinoxylans and arabinoxylans have been reported as the main polysaccharides of stem barks [4,5].

We then investigated the spasmolytic or spasmogenic effects of the different polysaccharide fractions on the spontaneous contractile activity of rat ileal smooth muscle. Indeed, alterations in smooth muscle contractility are among the key mechanisms involved in the pathophysiology of gastrointestinal disorders. To this end, pectic and hemicellulose fractions were applied to rat ileal smooth muscle. Pectic fractions from all the stem barks were found to induce a relaxation of the spontaneous contractile activity of the smooth muscle, indicating a myorelaxant activity. Lemon polygalacturonic acid, a commercially available, de-esterified homogalacturonan containing about 30% of RG-I, also induced a relaxation. In complement, pectic fractions from stem barks were demonstrated to be able to antagonize the smooth-muscle-stimulating action of acetylcholine, suggesting that they also exhibit antispasmodic properties. The structural motif of pectic polysaccharides responsible for the myorelaxant activity is questionable. The relative ratio between homogalacturonan and RG-I differs between pectins isolated from stem barks, with RG-I dominating in *A. klaineana* and *C. schweinfurthii* (a [GalA/Rha] ratio of about one) and homogalacturonans and RG-I being present in pectins of *S. zenkeri* and *P*. *butyracea* stem barks.

In contrast to pectins, the hemicellulose fractions extracted from the stem barks of the four plants, as well as wheat arabinoxylan and beechwood glucuronoxylan, induced an increase in the basal contractile activity of rat ileal smooth muscle. These results suggest that the spasmogenic activity of K1 and K4 hemicellulose fractions and of commercially available glucuronoxylan or arabinoxylan could be correlated to the presence of xylans in these fractions. In contrast, tamarind xyloglucan did not show any activity, confirming that xylans, instead of xyloglycans, exert an effect on the spontaneous contractile activity of ileal smooth muscle. K1 and K4 hemicellulose fractions differ both in their spasmogenic activity and in their Ara contents. Thus, this suggests that the level of Ara substitution of the xylan backbone may modulate the spasmogenic property of xylans. We also cannot rule out that the size of xylans may also affect their spasmogenic activity. Indeed, it has been reported that KOH enables the solubilization of polysaccharide populations of different sizes depending on the alkali concentration [17].

The alteration in smooth muscle contractility is among the main mechanisms involved in the pathophysiology of gastrointestinal disorders [15]. It is worth noting that several plant extracts have been reported to exhibit spasmolytic activity through their action as calcium channel blockers [15]. Indeed, the depressant effect of calcium channel blockage is known to impact the contraction of smooth muscles of the intestine and to affect cholinergic neurotransmission [18]. Data from the current study suggest that pectins and xylans may modulate the spontaneous contractile activity of rat ileal smooth muscle through their action on calcium or potassium channels. However, complementary studies have to be carried out to better define the size and structure of the minimal polysaccharide motifs responsible for these activities and their mechanisms of action on smooth muscles. This can be achieved by chemical or enzymatic degradation of backbones or side-chains of commercially available pectins or xylans and then the implementation of bioassays with these engineered polysaccharides, differing in length and composition, to investigate their antidiarrheal or laxative activity.

While the activity of some polysaccharides has been known for over 30 years, the lack of defined structural information has limited efforts to study their potential for clinical use. Some limitations may also occur for the therapeutic use of plant pectins and xylans against the symptoms of the irritable bowel syndrome. However, the use of newly available enzymes able to specifically hydrolyze glycosidic bonds of polysaccharide backbones or side-chains, as well as to remove glycan substitutions (*O*-acetyl or *O*-methyl groups), will greatly help in getting homogeneous polysaccharides to meet the requirements for their use as therapeutic agents for regulating intestinal motility.

## 4. Materials and Methods

### 4.1. Plant Material

The harvest of the stem bark from *Aucoumea klaineana*, *Canarium schweinfurthii* (Eudicots, Rosids, Malvids, Sapindales, Burseraceae), *Pentadesma butyracea* (Eudicots, Rosids, Fabids, Malpighiales, Clusiaceae) and *Scorodophloeus zenkeri* (Eudicots, Rosids, Fabids, Fabales, Fabaceae) [19] was carried in 2020 in Libreville (Gabon). Samples of these plants were deposited at the National Herbarium of Gabon (NHG), where they were identified by botanists from IPHAMETRA, Libreville, Gabon. Stem barks were dried out at room temperature for two weeks at the Department of Traditional Medicine of IPHAMETRA, Libreville, Gabon, and then reduced to a fine powder using a grinder.

### 4.2. Standard Polysaccharides

The sugar composition of the commercially available standard polysaccharides used in the present study were determined by analysis by gas chromatography with a flame ionization detector (GC-FID) to check their purity. Polygalacturonic acid from lemon (P-3850; Sigma-Aldrich, Saint-Quentin-Fallavier, France) consists of a linear chain of α(1-4)-linked GalA residues that are partially *O*-methylesterified. The detection of Rha (6%) and Gal (15%) in its sugar composition indicates that the sample also contains some RG-I, which was estimated to represent about 30% of the sample. Wheat arabinoxylan (P-WAXYM; Megazyme International Ireland) is a hemicellulose polymer composed of a β(1-4)-linked Xyl backbone substituted by α(1-3)-linked and α(1-2)-Ara residues. Wheat arabinoxylan is composed of 64% Xyl and 28% Ara. Beechwood glucuronoxylan (P-XYLNBE; Megazyme International Ireland) is composed of a β(1-4)-linked Xyl backbone substituted with α(1-2)-linked 4-*O*-methyl GlcA (4OMe-GlcA). Xyl is almost the only neutral monosaccharide detected in its sugar composition, which indicates that the sample is a pure glucuronoxylan. Xyloglucan from tamarind seeds (P-XYGLN; Megazyme International Ireland) is composed of a β(1-4)-linked Glc backbone that is substituted with α(1-6)-linked Xyl on three consecutive Glc units, the fourth residue being unsubstituted. Additional substitutions of Xyl with β(1-2)-linked Gal also occur. The sugar composition shows a ratio of 4/3/2 for Glc/Xyl/Gal, demonstrating that xyloglucan from tamarind is weakly contaminated by other polysaccharides.

### 4.3. Extraction of Pectin and Hemicelluloses

First, 500 g of stem barks was heated at 100 °C in 1 L of water for 1 h. After filtration, the insoluble residue was successively agitated in 1.6 L of methanol at 25 °C for 24 h and then in 3.7 L 70% ethanol at 70 °C for 1 h. The resulting alcohol insoluble residue was then heated in 2 L of 0.05% ammonium oxalate for 1 h at 100 °C. After filtration, the soluble fraction was dialyzed (Spectra/Por^®^ MWCO, 6-8000 Da; Fischer scientific, Illkirch, France) and freeze-dried to give the oxalate (Oxa) fraction. The insoluble residue was then treated with 1 M KOH containing 20 mM NaBH_4_ for 24 h at 25 °C. After centrifugation, the soluble fraction was dialyzed and freeze-dried as described above, to yield the K1 fraction. The same protocol was then applied to the K1 insoluble residue using 4 M KOH containing 20 mM NaBH_4_ to give the K4 fraction after centrifugation, dialysis and freeze-drying.

### 4.4. Monosaccharide Composition

The monosaccharide compositions of the bark polysaccharide extracts and commercially available standard polysaccharides were determined by gas chromatography–flame ionization detector (GC-FID) as previously reported [3].

### 4.5. Endo-Glucanase Digestion

About 10 mg of the K1 and K4 fractions was dissolved in 0.5 mL of 50 mM sodium acetate pH 5 and incubated at 37 °C for 18 h with 20 µL of endo-β(1,4)-glucanase (E-CELTR; Megazyme International Ireland, EC 3.2.1.4) from *Trichoderma* sp. Undigested polymers were then precipitated by addition of 2 mL of ethanol at −20 °C overnight. After centrifugation at 5000× *g* for 5 min, the ethanolic solution containing the xyloglucan fragments was air-dried.

### 4.6. Endo-Xylanase Digestion

About 10 mg of the K1 and K4 fractions was dissolved in 0.5 mL of 50 mM phosphate buffer pH 5 and incubated at 37 °C for 2 h with 20 µL (40 U) of endo-β(1,4)-xylanase endocellulase (xylanase M6, Megazyme International Ireland). Undigested polymers were then precipitated by addition of 2 mL of ethanol at −20 °C. After centrifugation at 5000× *g* for 5 min, the ethanolic solution containing the xyloglucan fragments was air-dried.

### 4.7. Oligosaccharide Analysis by Matrix-Assisted Laser Desorption/Ionization Time-of-Flight Mass Spectrometry (MALDI-TOF MS)

The mixture of oligosaccharides was analyzed by Matrix-Assisted Laser Desorption/Ionization time-of-flight mass spectrometry (MALDI-TOF MS). The mass spectra were recorded on a Bruker Daltonics MALDI-TOF device equipped with a 337 nm nitrogen laser. Freeze-dried oligosaccharides were dissolved in 50 μL of 0.1% trifluoroacetic acid in water. Then, 0.5 μL of this solution was added to 0.5 μL of the matrix solution (2, 5-dihydroxybenzoic acid at 20 mg/mL in 80% methanol). One µL of the resulting sample-matrix mixture was homogenized and deposited on the target. The different spectra were acquired in positive mode. Laser shots were accumulated for each spectrum (at least 10,000 laser shots per analysis). The analysis was performed using the Flex analysis software (v3.4).

### 4.8. In Vitro Assays on Excised Ileum Fragments

In vitro evaluation of the contractile activity and antispasmodic activities of polysaccharides on excised ileum fragments was performed according to the literature [3]. Pieces of ileum were dissected from Wistar rats and preserved during the tests in Mac Ewen’s physiological solution [in mM NaCl (130), KCl (5.63), CaCl_2_ (5.52), Na_2_HPO_4_ (0.93), NaHCO_3_ (11.9), MgCl_2_ (0.24) and glucose (11), pH 7.4]. Fragments measuring 0.5 to 0.9 cm were fixed in a tank, called a survival tank, in an aerated thermostatic bath at 37 °C. The basic activity (ileum contractions) of the organ or the stimulation of the organ with acetylcholine (10^−3^ µM) was recorded. Then, the organ was subjected to a single concentration of polysaccharides, loperamide or picosulphate (4 mg/mL). The response curves of ileum fragments to the plant extracts were recorded. The value of the amplitude before administration of the extracts or stimulation by acetylcholine was considered as a reference. The effects of the fractions on the intestinal spasms or those induced by acetylcholine were expressed as a percentage of inhibition = [(AB − AE)/AB] × 100, where AB is the average of the basal tone spasm or the stimulation with acetylcholine, and AE is the average of spasms in the presence of the extract or relaxation provoked by the extract on the contraction induced by acetylcholine. The spasmogene activity of fractions on the intestinal spasms were expressed as a percentage of stimulation = ((AE − AB)/AE) × 100, where AB is the average of the basal tone spasm, and AE is the average of spasms in the presence of the extract.

### 4.9. Data Analysis

The GraphPad Prism (version 8.4.3.686) and Excel package were used for the analyses. The results were presented as the mean ± standard deviation (SD) of *n* = 3 replications. Significance differences were established though One-way analysis of variance (ANOVA) followed by Dunnett’s multiple comparison test.

## 5. Conclusions

Pectin is usually considered as an antidiarrheic polymer because this water-*soluble fiber* can absorb water, leading to an increase in stool consistency. In contrast, hemicelluloses are insoluble fibers that induce the secretion of water. In the present study, we show that the properties of these polysaccharides on the affections of irritable bowel syndrome are not restricted to these physicochemical effects but are also due to their activities on the spontaneous contractile activity of smooth muscles. The current study shows that pectic polysaccharides isolated from barks of four Gabonese plants and lemon polygalacturonic acid exhibit myorelaxant activities, whereas hemicellulosic fractions from the same barks, as well as wheat arabinoxylan and beechwood glucuronoxylans, display spasmogenic activities. Further studies will have to be performed to determine the minimal pectic and xylan motifs responsible for the observed outcomes on the spontaneous contractile activity of smooth muscle and to determine their mechanisms of action.

## Figures and Tables

**Table 1:** Extraction yields of pectins and hemicelluloses from stem barks of *A. klaineana*, *C. schweinfurthii*, *P. butyracea* and *S. zenkeri*.

Plant	Code	Extract	Yield (%)	% Pectins and Hemicelluloses
*Aucoumea klaineana*	AKoxa	Oxalate	0.1	1.5
	AKK1	1 M KOH	3	98.5
	AKK4	4 M KOH	4	
*Pentadesma butyracea*	PBoxa	Oxalate	0.4	3.7
	PBK1	1 M KOH	4.1	96.3
	PBK4	4 M KOH	6.1	
*Canarium schweinfurthii*	CSoxa	Oxalate	0.05	1
	CSK1	1 M KOH	1.8	99
	CSK4	4 M KOH	2.8	
*Scorodophloeus zenkeri*	SZoxa	Oxalate	0.3	4.6
	SZK1	1 M KOH	2.1	95.4
	SZK4	4 M KOH	4	

**Table 2:** The sugar composition of pectin fractions isolated by oxalate treatment of stem barks of *Aucoumea klaineana* (AKoxa), *Pentadesma butyracea* (PBoxa), *Canarium schweinfurthii* (CSoxa) and *Scorodophloeus zenkeri* (SZoxa). The results are presented as the mean ± standard deviation of three independent experiments.

Monomer	AKoxa	PBoxa	CSoxa	SZoxa
Ara	18.3 ± 3	32.9 ± 6	30.4 ± 6	14.1 ± 3
Rha	8.8 ± 2	7.7 ± 2	11 ± 2	9.4 ± 2
Fuc	2.0 ± 0.8	0.6 ± 0.2	1.4 ± 0.5	2.0 ± 0.5
Xyl	8.7 ± 2	10.2 ± 3	9.6 ± 3	8.3 ± 2
GlcA	4.2 ± 2	3.0 ± 1	3.1 ± 1	2.7 ± 1
Man	5.5 ± 2	3 ± 1	4.4 ± 1	5.5 ± 2
Gal	38.8 ± 7	24.2 ± 6	35.9 ± 7	13.9 ± 3
GalA	9.9 ± 3	17.4 ± 5	13 ± 3	39.8 ± 7
[GalA/Rha]	1.13	2.25	1.18	4.23
[GalA/Ara]	2.12	0.8	1.17	0.96
[(Gal+Ara)/Rha]	6.48	7.3	6.02	2.98

**Table 3:** The sugar composition of hemicelluloses isolated by KOH treatments. The results are presented as the mean ± standard deviation. *n* = 3.

Monomer	*Aucoumea klaineana*		*Pentadesma butyracea*		*Canarium schweinfurthii*		*Scorodophloeus zenkeri*	
	AKK1	AKK4	PBK1	PBK4	CSK1	CSK4	SZK1	SZK4
Ara	5.6 ± 1	6.5 ± 1	25.2 ± 3	12.5 ± 2	10.5 ± 2	6.3 ± 1	6.7 ± 1	8.4 ± 1
Rha	2.0 ± 0.5	3.0 ± 1	3.0 ± 0.5	2.1 ± 0.5	2.8 ± 0.1	1.0 ± 0.1	2.5 ± 0.5	1.1 ± 0.2
Fuc	0.8 ± 0	1.0 ± 0.2	0.6 ± 0.1	1.5 ± 0.2	0.9 ± 0.2	0.8 ± 0.2	1.9 ± 0.5	1.0 ± 0
Xyl	55.9 ± 5	42.9 ± 4	29.4 ± 3	49.5 ± 5	22.6 ± 3	65.4 ± 5	60.8 ± 7	75.1 ± 5
GlcA	2.1 ± 0.2	2.3 ± 0.7	1.9 ± 0.3	1.6 ± 0.1	2.6 ± 0.2	0.7 ± 0.1	1.6 ± 0.2	0.7 ± 0.1
Man	1.4 ± 0.2	2.6 ± 0.5	1.7 ± 0.2	3.9 ± 0.5	1.6 ± 0.2	1.7 ± 0.2	3.8 ± 0.1	1.3 ± 0.1
Gal	4.4 ± 0.4	11.1 ± 2	17.3 ± 3	10.8 ± 2	8.5 ± 1	2.9 ± 0.5	4.9 ± 0.4	2.7 ± 0.5
GalA	2.2 ± 0.2	6.2 ± 0.3	10.6 ± 2	3.8 ± 0.5	5.0 ± 0.5	1.3 ± 0.2	5.7 ± 0.5	2.6 ± 0.3
Glc	25.7 ± 4	23.3 ± 3	11.4 ± 2	13.9 ± 3	45 ± 5	19.7 ± 2	12.1 ± 2	6.7 ± 0.5
Xyl/Ara	10	6.6	1.1	4	2	10	9	9

**Table 4:** Myorelaxant and antispasmodic activities of pectic fractions and loperamide tested at 4 mg/mL on the smooth muscle. **** *p* < 0.0001 by comparison to loperamide. ^###^ *p* < 0.001 and ^####^ *p* < 0.0001 by comparison to acetylcholine.

	Extracts	% Relaxation ± SD ^a^
Positive control	Loperamide	55 ± 4
Plant extracts	AKoxa	100 ± 0 ****
	CSoxa	100 ± 0 ****
	PBoxa	59.0 ± 9.0
	SZoxa	52.5 ± 4.1
Standard polysaccharide	Lemon polygalacturonic acid	100 ± 0 ****
Acetylcholine (10^−3^ µM) + plant extracts ^b^	Acetylcholine (10^−3^ µM) + PBoxa	41.9 ± 4 ^####^
	Acetylcholine (10^−3^ µM) + SZoxa	59.1 ± 9 ^###^

^a^ SD: Standard deviation of three independent experiments. *n* = 3. ^b^: AKoxa and CSoxa pectic extracts were not isolated in a sufficient amount to perform these assays.

**Table 5:** Activity of polysaccharides extracted from *A. klaineana*, *C. schweinfurthii*, *P. butyracea* and *S. szenkeri*; commercially available wheat arabinoxylan, beechwood glucuronoxylan and tamarind xyloglucan; and picosulphate tested at 4 mg/mL on the stimulation of the contractile activity of rat ileal fragments. ** *p* < 0.01, *** *p* < 0.001 or **** *p* < 0.0001 by comparison picosulphate.

	Sample	% Stimulation ± SD ^a^
Positive control	Picosulphate	84.0 ± 1.3
Plant extracts	AKK1	205.3 ± 14.2 ****
	CSK1	277.9 ± 28.2 ****
	PBK1	165.0 ± 21.1 **
	SZK1	195.5 ± 3.3 ***
	AKK4	189.0 ± 7.0 ***
	CSK4	366.6 ± 10.5 ****
	PBK4	411.1 ± 38.6 ****
	SZK4	363.3 ± 15.2 ****
Standard polysaccharides	Wheat arabinoxylan	94.2 ± 10.7
	Beechwood glucuronoxylan	61.8 ± 3.7
	Tamarind xyloglucan	0 ± 0

^a^ SD: Standard deviation of three independent experiments. *n* = 3.

## Data Availability

All data are presented in the article.

## Supplementary Materials

The following supporting information can be downloaded at: https://www.mdpi.com/article/10.3390/molecules30153156/s1. Figure S1: Recordings of the contractile activity of smooth muscle after addition (arrows) of spasmolytic (polygalacturonic acid (APGal), CSoxa) or spasmogenic fractions (AKK1, PBK4).

## Abbreviations

The following abbreviations are used in this manuscript:

RG-I	rhamnogalacturonan I
Oxa	pectins extracted with oxalate
K1 and K4	hemicelluloses extracted with 1 M and 4 M KOH
AK	*Aucoumea klaineana*
PB	*Pentadesma butyracea*
CS	*Canarium schweinfurthii*
SZ	*Scorodophloeus zenkeri*
4OMe-GlcA	4-*O*-methyl glucuronic acid
GC-FID	gas chromatography–flame ionization detector
MALDI-TOF MS	Matrix-Assisted Laser Desorption/Ionization time-of-flight mass spectrometry

## References

[1] Yu Y., Shen M., Song Q., Xie J. (2018). Biological activities and pharmaceutical applications of polysaccharide from natural resources: A review. Carbohydr. Polym..

[2] Minzanova S.T., Mironov V.F., Arkhipova D.M., Khabibullina A.V., Mironova L.G., Zakirova Y.M., Milyukov V.A. (2018). Biological activity and pharmacological application of pectic polysaccharides: A review. Polymers.

[3] Mayindza Ekaghba E.L., Loutelier-Bourhis C., Schmitz I., Afonso C., Lerouge P., Mengome L.E. (2024). Phytochemical analysis and antidiarrheal activity of bark decoctions of Pentadesma butyracea Sabine (Clusiaceae). Molecules.

[4] Gowda D., Sarathy C. (1987). Structure of an L-arabino-D-xylan from the bark of *Cinnamomum zeylanicum*. Carbohydr. Res..

[5] Kanari M., Tomoda M., Gonda R., Shimizu N., Kimura M., Kawaguchi M., Kawabe C.A. (1989). A reticuloendothelial system-activating arabinoxylan from the bark of *Cinnamomum cassia*. Chem. Pharm. Bull..

[6] Fradinho D.M., Pascoal Neto C., Evtuguin D., Jorge F.C., Irle M.A., Gil M.H., Pedrosa de Jesus J. (2002). Chemical characterisation of bark and of alkaline bark extracts from maritime pine grown in Portugal. Ind. Crops Prod..

[7] Mengome L.E., Voxeur A., Akue J.P., Lerouge P. (2014). In vitro proliferation and production of cytokine and IgG by human PBMCs stimulated with polysaccharide extract from plants endemic to Gabon. Molecules.

[8] Ferreira J.P.A., Miranda I., Sousa V.B., Pereira H. (2018). Chemical composition of barks from *Quercus faginea* trees and characterization of their lipophilic and polar extracts. PLoS ONE.

[9] Flint H., Bayer E., Rincon M., Lamed R., White B.A. (2008). Polysaccharide utilization by gut bacteria: Potential for new insights from genomic analysis. Nat. Rev. Microbiol..

[10] Liu B., Zhang Z., Liu X., Hu W., Wu W. (2023). Gastrointestinal fermentable polysaccharide is beneficial in alleviating loperamide-induced constipation in mice. Nutrients.

[11] Combo A.M.M., Aguedo M., Paquot M. (2011). Les oligosaccharides pectiques: Production et applications possibles. Biotechnol. Agron. Soc. Environ..

[12] Huang J., Lin B., Zhang Y., Xie Z., Zheng Y., Wang Q., Xiao H. (2022). Bamboo shavings derived O-acetylated xylan alleviates loperamide-induced constipation in mice. Carbohydr. Polym..

[13] Vyshtakalyuk A.B., Sosnina N.A., Minzanova S.T., Zobov V.V., Lantsova A.V., Minullina E.R., Mironov V.F., Karaseva A.N. (2006). Effect of pectin substances on contractile activity of the uterine myometrium in rats. Bull. Exp. Biol. Med..

[14] Graça J., Bezerra M., Lima V., Rodrigues J., Monteiro D., Quinderé A., Amorim R., Paula R., Benevides N. (2011). Effect of a Crude Sulfated polysaccharide from *Halymenia floresia* (Rhodophyta) on gastrointestinal smooth muscle contractility. Braz. Arch. Biol. Technol..

[15] Rawat P., Singh P.K., Kumar V. (2017). Evidence based traditional anti-diarrheal medicinal plants and their phytocompounds. Biomed. Pharmacother..

[16] Mougnala Moukagni E., Ziegler-Devin I., Safou-Tchima R., Brosse N. (2021). Extraction of acetylated glucuronoxylans and glucomannans from Okoume (*Aucoumea klaineana* Pierre) sapwood and heartwood by steam explosion. Ind. Crops Prod..

[17] Xie Y., Guo X., Ma Z., Gong J., Wang H., Lv Y. (2020). Efficient extraction and structural characterization of hemicellulose from sugarcane bagasse Pith. Polymers.

[18] De Ponti F., Giaroni C., Cosentino M., Lecchini S., Frigo G. (1993). Calcium-channel blockers and gastrointestinal motility: Basic and clinical aspects. Pharmacol. Ther..

[19] Angiosperm Phylogeny Group (2009). An update of the Angiosperm Phylogeny Group classification for the orders and families of flowering plants. Bot. J. Linn. Soc..

