# Supplementary material for: Effects of Polysaccharides Extracted from Stem Barks on the Spontaneous Contractile Activity of the Ileal Smooth Muscle
Source: Molecules. 2025 Jul 28;30(15):3156. doi: 10.3390/molecules30153156 (PMC12348852; doi:10.3390/molecules30153156)
Supplement: Supplementary file 1 [file molecules-30-03156-s001.zip › molecules-3751538-supplementary.pdf]

Supplementary Material

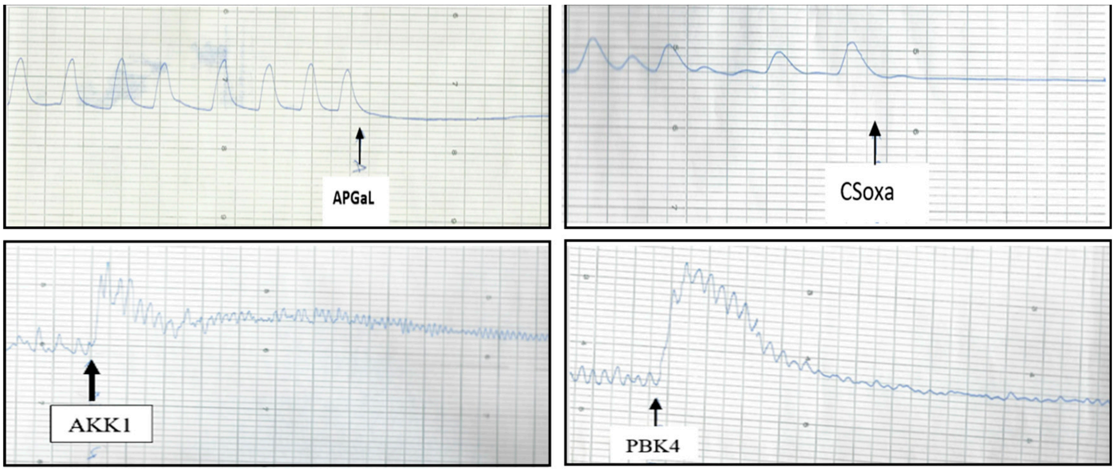

Figure S1. Recordings of the contractile activity of smooth muscle after addition (arrows) of spasmolytic (polygalacturonic acid (APGal), CSoxa) or spasmogenic fractions (AKK1, PBK4).
